# Lentivirus Mediated siRNA against GluN2B Subunit of NMDA Receptor Reduces Nociception in a Rat Model of Neuropathic Pain

**DOI:** 10.1155/2014/871637

**Published:** 2014-08-28

**Authors:** Feixiang Wu, Ruirui Pan, Jiaying Chen, Megumi Sugita, Caiyang Chen, Yong Tao, Weifeng Yu, Yuming Sun

**Affiliations:** ^1^Department of Anesthesiology, Eastern Hepatobiliary Hospital, Second Military Medical University, Shanghai 200438, China; ^2^Department of Anesthesiology and Critical Care, Perelman School of Medicine at University of Pennsylvania, Philadelphia, PA 19104, USA

## Abstract

Although neuropathic pain (NP) is still not fully understood by scientists and clinicians alike, studies suggest that N-methyl-D-aspartate (NMDA) receptors play an important role in the induction and maintenance of NP. A promising treatment for NP is through the downregulation of NMDA subunit GluN2B by RNA interference; however, naked siRNA (small interference RNA) is not effective in long-term treatments. In order to concoct a viable prolonged treatment for NP, Lv-siGluN2B (lentivirus carrying siRNA targeting GluN2B subunit) was prepared and the antinociception effects were observed in chronic constriction injury (CCI) rats in the present study. Results showed that Lv-siGluN2B was transduced into spinal cord cells after intrathecal injections and effectively reduced the nociception induced by sciatic nerve ligation while inhibiting the mRNA and protein expression of GluN2B. This antinociception effect lasted approximately 7 weeks. These findings suggest that GluN2B subunit could be a target for NP treatment and Lv-siGluN2B represents a new potential option for long-term treatment of NP.

## 1. Introduction

Neuropathic pain (NP) is characterized by hyperalgesia, allodynia, and spontaneous pain. It often occurs as a result of injury to peripheral nerves, dorsal root ganglions (DRG), spinal cord, or brain. An estimated 7% to 8% of the general population suffers from mild to moderate forms of NP, and 5% may be severely affected by it [1, 2]. The N-methyl-D-aspartate (NMDA) receptor activation in animal models of chronic pain has implicated that NMDA receptors affect the polysynaptic spinal pathways and chronic nociceptive responses [3–5]. The current study focuses on the intrathecal administration of GluN2B (formally named NR2B) subunit of NMDA, which may evoke a selective, dose-dependent, and reversible hyperalgesia in mice and rats [6].

It is well documented in clinical and experimental cases that NMDA receptor agonists profoundly inhibit the long-term potentiation in the spinal cord [7]; however, their use as analgesics is limited by serious side effects [8, 9]. Currently, the available pharmacological NMDA receptor antagonists are nonspecific for NMDA receptor subtypes, but with the ever increasing knowledge of RNA interference (RNAi) and small interfering (siRNA), it is plausible to develop novel drugs that target or knock out genes for the treatment of chronic pain [10, 11]. Tan et al. [12] reported that GluN2B receptor, knocked down by intrathecal injection of siRNA, could reduce formalin-induced nociception in rats for approximately 21 days [12]. Song et al. [13] also demonstrated that mRNA levels in siRNA-treated mice were only 40% of those in control mice on day 14 and returned to normal on day 20 after the last injection. Our prior study investigated the silencing effect of naked siRNA targeting Toll-like receptor (TLR), which lasted only 3 days after the last injection [14]. For this reason, we considered alternative mechanisms for long-term treatment of NP.

Lentiviral vector allows for sustained transgene delivery, nondividing and dividing cells infection, and broad tissue tropism, making it a more efficient and safer vehicle for spinal cord transduction [15]. Experimental transduction in neurons and glia cells of mice and rats after intraparenchymal injection displayed therapeutic effects lasting over 4 weeks [16, 17]. We hypothesize that the long-term treatment of NP can be achieved through utilizing lentivirus with siRNA targeting GluN2B receptors, and the antinociception effect can be observed in chronic constriction injury (CCI) rats.

## 2. Materials and Methods

### 2.1. Production and Identification of Recombinant Lentivirus Lv-siGluN2B

The siRNA (CCTGTGTGCCTAACAACAA) targeting GluN2B subunit of NMDA receptor gene (GenBank accession NM 000834) was screened and tested as described in our previous study [14]. Based on the sequences of lentivirus and the “Tuschl” principle, target sequences were designed and chemically synthesized in United Gene Company (Shanghai, China) and were under control of U6 promoter in lentivirus. The green fluorescent protein (GFP) was also addressed in the lentivirus to detect the transfected location of the lentivirus after intrathecal injection with* Hpa* I and* Xho* I restriction sites at the 5′ and 3′ ends, respectively. After pFU-GW-siRNA was digested by* Hpa* I and* Xho* I (TaKaRa, Japan), target gene was cloned into pFU-GW-siRNA and named pFU-GW-siGluN2B. To produce recombinant lentivirus Lv-siGluN2B (lentivirus-expressing siRNA of GluN2B), pFU-GW-si GluN2B (20 *μ*g), pHelper 1.0 (15 *μ*g), and pHelper 2.0 (10 *μ*g) were cotransfected into HEK 293T cells with Lipofectamine 2000. Lentivirus was harvested at about 48 h after transfection. The final titer of recombinant virus was adjusted to 1 × 10^9^ TU/mL.

### 2.2. Animals and Chronic Constriction Injury (CCI)

Male Sprague-Dawley (SD) rats weighing 200–250 g were obtained from Shanghai Experimental Animal Center, the Chinese Academy of Sciences. The CCI model was established as previously described [18]. Briefly, after rats were anesthetized with sodium pentobarbital (40 mg/kg, i.p.), the right sciatic nerve was exposed at the mid-thigh level. The nerve was ligated loosely with 4-0 chromic gut threads at 4 sites with 1 mm apart, so that the nerve diameter was only slightly reduced. In the sham group, the sciatic nerve was exposed without ligation. Upon recovery from anesthesia, animals were housed individually in clear plastic cages. All animal experiments were approved by the Administrative Committee of Experimental Animal Care and Use of Second Military Medical University and conformed to the National Institute of Health guidelines on the ethical use of animals.

### 2.3. Lumbar Subarachnoid Catheterization

Rats were implanted with chronic indwelling catheters in the subarachnoid space on the same day after CCI procedure. Briefly, rats were anesthetized with sodium pentobarbital (40 mg/kg, i.p.). A PE-10 catheter (Becton Dickinson, Sparks, MD, USA) was inserted into the lumbar subarachnoid space between lumbar vertebrae 5 (L5) and L6 [19]. The catheter was chronically implanted and the external part of the indwelling catheter was protected according to Milligan's method [20]. A lidocaine test was given to determine the functionality and position of the catheter tip in the subarachnoid space.

### 2.4. Intrathecal Delivery of Lentivirus

Rats were randomly divided into 4 groups (*n* = 90 per group): sham group (sham surgery + normal saline), normal saline (NS) group (CCI + NS), Lv-GFP group (CCI + Lv-GFP), and Lv-siGluN2B group (CCI + Lv-siGluN2B). Lentivirus Lv-GFP expressing scrambled siRNA (TTCTCCGAACGTGTCACGT) was used as a control. After confirmation of the effect of CCI on the 3rd day after surgery, rats in Lv-GFP group and Lv-siGluN2B group were given Lv-GFP and Lv-siGluN2B (1 × 10^7^ TU/10 *μ*L), respectively. The normal saline of equal volume was administered intrathecally in rats of the remaining two groups.

### 2.5. Evaluation of Thermal Hyperalgesia

The paw withdrawal latency (PWL) to radiant heat was used to evaluate the thermal hyperalgesia as previously described [21]. The PWL was measured on the day and on the 1st, 3rd, 7th, 10th, 14th, 21st, 28th, and 35th days after intrathecal injection of the virus. Rats were placed under an inverted clear plexiglass cage (23 × 18 × 13 cm) on a piece of 3 mm thick glass plate and were allowed to acclimate to the surroundings for 30 min before testing. Then, the radiant heat source was positioned under the glass floor directly beneath the right hind paw. The radiant heat source consisted of a high-intensity projection lamp bulb (8 V, 50 W), locating 40 mm below the glass floor and projecting through a 5 × 10 mm aperture at the top of a movable case. A digital timer automatically read the time from stimuli to PWL. Detection was done twice in each rat with a 5-minute interval. The cut-off time was set at 20 sec to avoid tissue damage.

### 2.6. Evaluation of the Tactile Allodynia

The paw withdrawal threshold (PWT) was used to evaluate the mechanical allodynia for pain. Mechanical allodynia was assessed with von Frey filaments on the day and on the 1st, 3rd, 7th, 10th, 14th, 21st, 28th, and 35th days after intrathecal injection of the virus. Rats were placed on a wire mesh platform, covered with a transparent plastic dome, and allowed to acclimate for 30 min before testing. The filament was applied perpendicularly to the plantar surface of the right hind paw. The PWT was determined by sequentially increasing and decreasing the stimulus strength (the “up-and-down” method) (in gram, g), and data were analyzed using the nonparametric method of Dixon [22].

### 2.7. Spinal Cord RNA Extraction and Real-Time PCR

The real-time PCR was performed on the day and on the 1st, 3rd, 7th, 10th, 14th, 21st, 28th, and 35th days after intrathecal injection of the virus. Total RNA (6 samples of each group) was extracted from L4-L5 spinal cord. Extracted RNA was treated with DNase I at 37°C for 30 min before reverse transcription was performed using a kit (TaKaRa, Japan). The PCR primers were as follows: 5-CGGGAG CTC TGA ATG CTC TCT TGC ATC TGG CTG GC-3 (forward) and 5-CGG GTC GAC GCC ATA CAA TTC GACCTG CTG-3 (reverse). The Real-Time PCR Detection System (Roche, Switzerland) continually monitors the increase in fluorescence, which is directly proportional to the PCR product.

### 2.8. Western Blot Assay

The proteins of tissues were prepared from lumbar spinal cord (L4-L5) on the 7th day after injection as previously described [23]. Proteins were separated by 8% polyacrylamide SDS-PAGE and transferred onto a nitrocellulose membrane. The nitrocellulose membrane was blotted with a primary antibody against GluN2B subunit of NMDA (1 : 100, RayBiotech, USA) and then with secondary antibody conjugated with horseradish peroxidase. Protein signals were detected with an ECL system (Amersham Pharmacia, Uppsala, Sweden). GAPDH (Sigma Chemical Co., MO, USA, 1 : 500) was used as a loading control.

### 2.9. Immunofluorescence Assay

Rats were anesthetized and perfused through the ascending aorta with NS and then with 4% paraformaldehyde in 0.16 M phosphate buffer (pH 7.2–7.4) containing 1.5% picric acid. After perfusion, the L5 spinal cord was collected and fixed in the same fixation solution for 3 h and then in 15% sucrose overnight. Transverse spinal sections (30 *μ*m) were obtained on a cryostat and processed for immunofluorescence assay [24]. All the sections were blocked in 0.3% Triton X-100 containing 2% goat serum for 1 h at room temperature and incubated over two nights at 4°C with anti-GluN2B antibody (1 : 400; RayBiotech, USA). The sections were incubated for 1 h at room temperature with Cy3-conjugated secondary antibody (1 : 300; Santa Cruz, USA). These sections were examined under an Olympus (Olympus, Japan) fluorescence microscope, and representative images were captured.

### 2.10. Statistical Analysis

All data were expressed as mean ± standard error (SEM). Statistical analysis was carried out using two-way ANOVA followed by Turkey's multiple comparisons using GraphPad Prism software (Version 5, GraphPad Software Inc., CA, USA). The image data from western blotting was compared using one-way ANOVA. A value of *P* < 0.05 was considered statistically significant.

## 3. Results

### 3.1. Transfection of Neurocytes by Lentivirus

**Figure 1:**
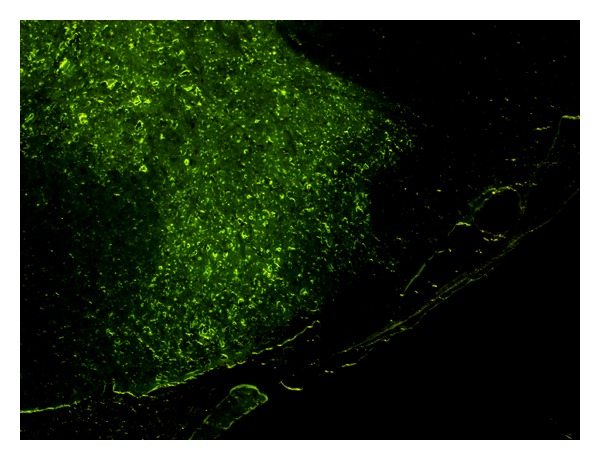
Detection of lentivirus Lv-siGluN2B by GFP expression (×100). As shown in Figure 1, GFP positive cells were observed in the spinal cord of the rats in Lv-siGluN2B group after intrathecal injection, which suggested that the lentivirus was efficiently transduced into cells of the spinal cord.

The location of the lentivirus could be tracked by GFP expression due to the lentiviral vector system. As shown in Figure 1, the lentivirus was efficiently transduced into cells of the spinal cord. The fluorescence of GFP was observed in the neurocytes of rats in Lv-siGluN2B group.

### 3.2. Lv-siGluN2B Decreased GluN2B Expression in CCI Rats

**Figure 2:**
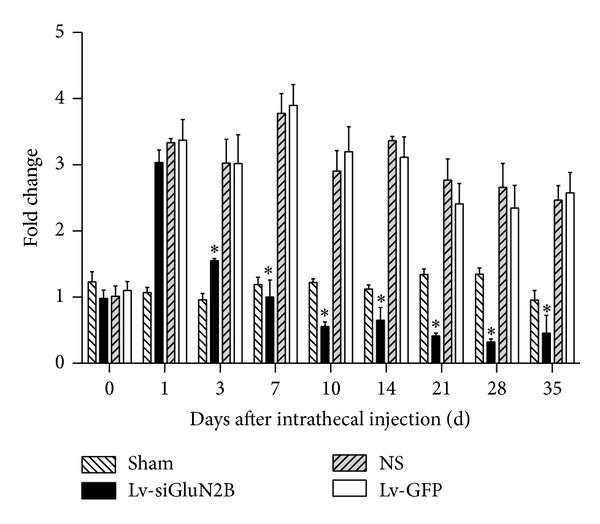
mRNA expression of GluN2B detected by real-time PCR. As shown in Figure 2, CCI increased the GluN2B mRNA expression in Lv-siGluN2B group, Lv-GFP group, and NS group. On the 3rd, 7th, 10th, 14th, 21st, 28th, and 35th days after delivery of Lv-siGluN2B, the GluN2B mRNA expression induced by nerve ligation decreased significantly compared to Lv-GFP group and NS group (*P < 0.01 versus NS and Lv-GFP groups, two-way ANOVO analysis followed by Turkey's multiple comparisons, *N* = 6). No differences were observed on the 1st day.

**Figure 3:**
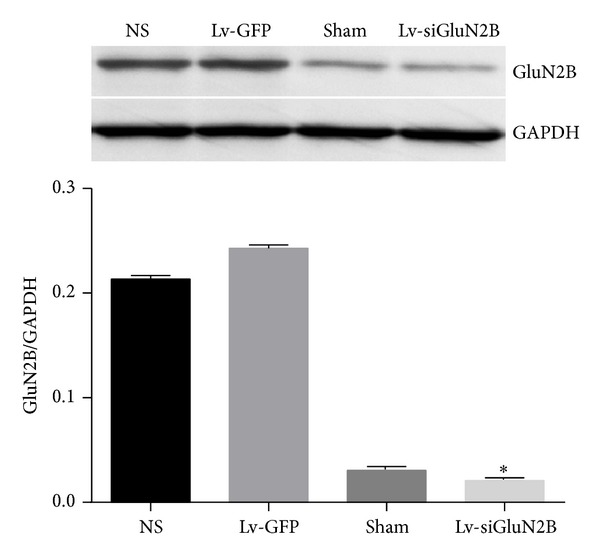
Western blot assay of GluN2B expression. The protein expression of GluN2B was also markedly downregulated, which was confirmed by western blot assay (*P < 0.01 versus NS and Lv-GFP group, one-way ANOVO analysis, *N* = 6).

**Figure 4:**
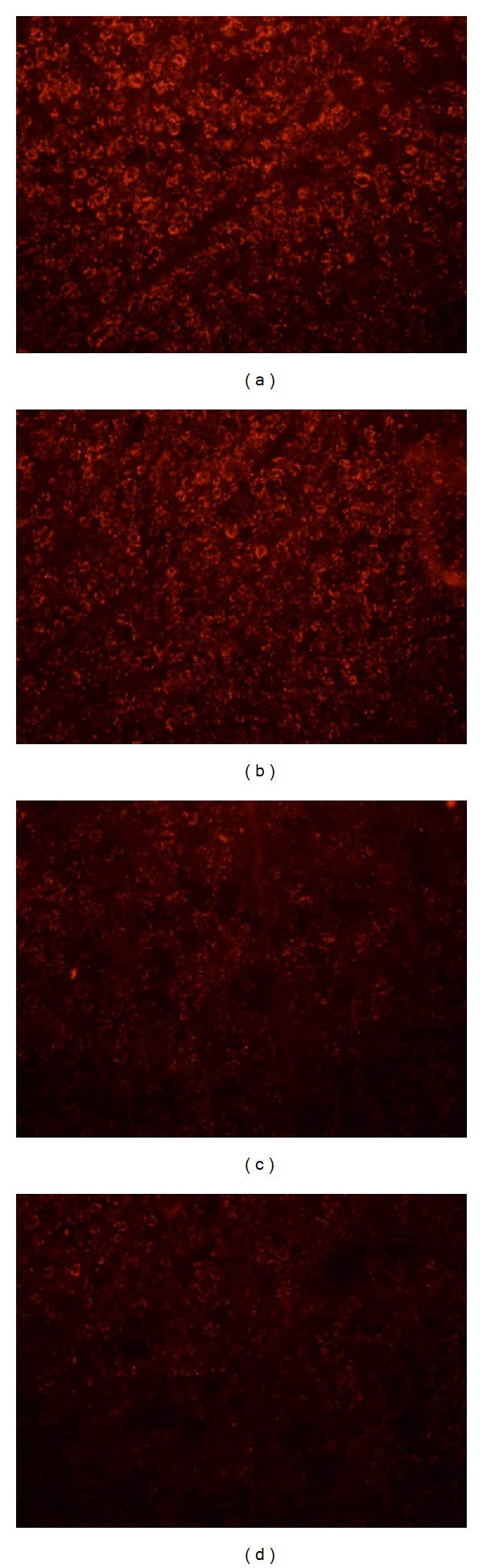
Immunofluorescence staining of GluN2B. Downregulation of GluN2B subunit in Lv-siGluN2B group was corroborated with findings in immunohistostaining in which the GluN2B-positive cells in the spinal cord were detected. (a) NS (×100). (b) Lv-GFP (×100). (c) Sham (×100). (d) Lv-siGluN2B (×100).

Lv-siGluN2B was intrathecally delivered into CCI rats and the expression of GluN2B was detected. As shown in Figure 2, CCI increased the mRNA expression of GluN2B. Compared to the sham group, GluN2B mRNA expression increased significantly in NS and Lv-GFP groups (*P* < 0.01, two-way ANOVO analysis followed by Turkey's multiple comparisons, *N* = 6). Three days after delivery of Lv-siGluN2B, the mRNA expression of GluN2B induced by nerve ligation decreased significantly (*P* < 0.01 versus NS and Lv-GFP groups, *N* = 6). Similarly, western blot assay (Figure 3) showed that the protein expression of GluN2B was increased in NS and Lv-GFP groups after ligation and downregulated by the Lv-siGluN2B (*P < 0.01 versus NS and Lv-GFP groups, one-way ANOVO analysis, *N* = 6). These changes correspond with the results of immunohistostaining in the spinal cord, where GluN2B-positive cells were detected (Figure 4). Following ligation, the GluN2B expression dramatically increased in NS group, while the GluN2B expression significantly decreased in Lv-siGluN2B group but not in LV-GFP group.

### 3.3. Lv-siGluN2B Attenuated NP in CCI Rats for 35 Days

**Figure 5:**
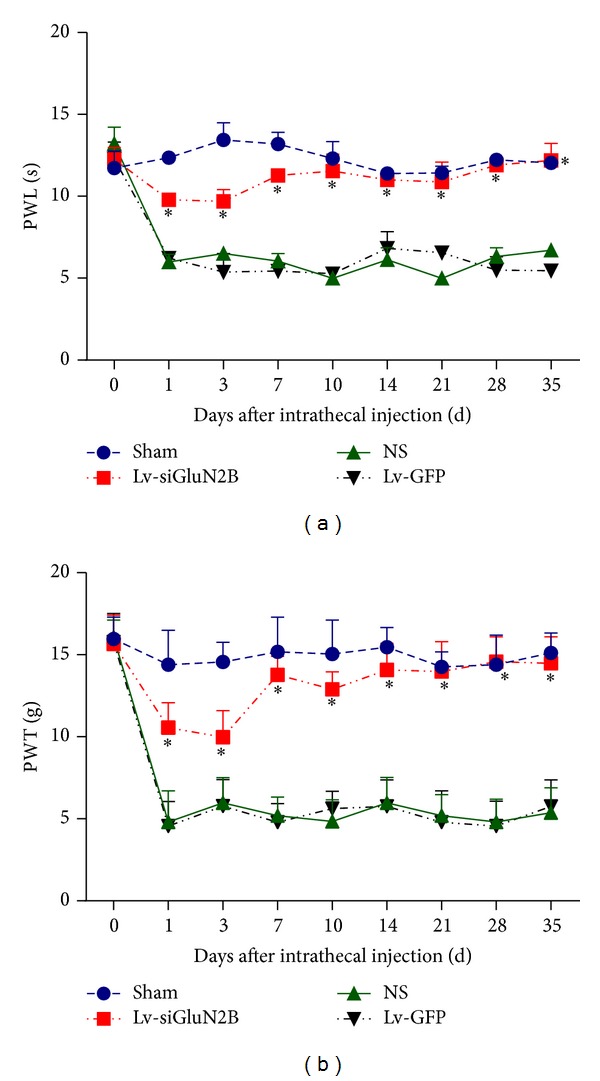
Impact of Lv-siGluN2B on PWL and PWT in CCI rats. On the 1st, 3rd, 7th, 10th, 14th, 21st, 28th, and 35th days after intrathecal injection, CCI rats receiving intrathecal Lv-siGluN2B showed significantly attenuated thermal hyperalgesia (a) and mechanical allodynia (b) compared to CCI rats treated with Lv-GFP and NS, as showed by PWL (*P < 0.01 versus NS and Lv-GFP groups, two-way ANOVO analysis followed by Turkey's multiple comparisons, *N* = 10) and PWT (*P < 0.01 versus NS and Lv-GFP groups, two-way ANOVO analysis followed by Turkey's multiple comparisons, *N* = 10).

To examine the impact of Lv-siGluN2B on pain response* in vivo*, modulation of pain perception in the Bennett model of NP was investigated. PWT and PWL were used to measure the mechanical allodynia and thermal hyperalgesia, respectively. After surgery, pain response such as mechanical allodynia and thermal hyperalgesia was induced, in correspondence to the reduced PWL and PWT. CCI rats receiving intrathecal Lv-siGluN2B showed significantly attenuated mechanical allodynia and thermal hyperalgesia (*P* < 0.01 versus NS and Lv-GFP groups, two-way ANOVO analysis followed by Turkey's multiple comparisons, *N* = 10), in contrast to CCI rats treated with Lv-GFP (Figure 5). The attenuation of pain response was NMDA specific since CCI rats receiving Lv-GFP intrathecally had no pain relief, as compared to NS treated CCI rats. The process lasted for about 35 days, which suggests that the anti-NP effect of Lv-siGluN2B was long lasting.

## 4. Discussion

In this study, the effects of NP on rat CCI models were investigated using constructed lentivirus-expressing siRNA against GluN2B subunit of NMDA receptors. Based on our results, not only could Lv-siGluN2B be successfully transfected into the spinal cord by intrathecal injection, but also its induction significantly downregulated mRNA and protein expression of GluN2B in the spinal cord. In addition, Lv-siGluN2B was effectively attenuated in the CCI-induced thermal and mechanical pain hypersensitivity for about 7 weeks. Our findings suggest that the lentivirus-mediated siRNA against GluN2B may be used for gene therapy of NP in an experimental setting. If this is successful, the downregulation of GluN2B expression by Lv-siGluN2B may be used to treat NP.

Many studies have shown that GluN2B is distributed throughout the spinal cord and plays an important role in the formation of central sensitization and persistent pain [25, 26]. GluN2B-NMDA receptor activation exacerbates a range of Ca^2+^-sensitive signaling cascades, leading to an enhancement of responsiveness to synaptically released glutamate [27]. This increase in responsiveness leads to pain hypersensitivity. In the present study, GluN2B was found to be upregulated in RNA and protein levels after sciatic nerve ligation, consistent with the previous studies in different chronic pain models [28–30], which suggests the possibility of using this mechanism as a target for the NP treatment. Previous behavioral studies have shown that systematical application of the selective GluN2B antagonists, ifenprodil and CP-101,606, produce analgesia in animals with persistent inflammatory or neuropathic pain [29, 30]. Intrathecal (i.t.) injection of Ro 25-6981, a selective GluN2B antagonist, had a dose-dependent antiallodynic effect that leaves motor functions in tact [31]. In the present study, Lv-siGluN2B decreased the mRNA and protein expression of GluN2B along with the attenuation of hyperalgesia and mechanical allodynia. Thus, the spinal cord GluN2B is a novel target for NP treatment.

In the current study, RNAi was used as a powerful technique to “knock down” target gene of GluN2B. RNAi utilizes the ability of double-stranded RNAs (dsRNA) allowing it to form RNA duplexes of specific length and structure for the purpose of guiding the degradation of mRNA homologous sequences to siRNA and inducing sequence-specific gene silencing* in vivo* [32]. One of the potential advantages of this technology is the ability to design precisely targeted therapeutics of any specific subtype [33]. The NMDA receptor is composed of the GluN1 and GluN2 subunits (GluN2A, GluN2B, GluN2C, and GluN2D). The GluN2 subunits determine the characteristics of NMDAR channels by forming different heteromeric configurations with the GluN1 subunit [34]. In the present study, only the GluN2B subunit of NMDA was specifically targeted by the Lv-siGluN2B. The GluN2B expression was downregulated by the Lv-siGluN2B, while the Lv-GFP expressing scrambled siRNA showed no effect on GluN2B expression. The mRNA expression of Lv-siGluN2B treated group decreased up to 75%. siRNA targeting P2X3, *δ*-opioid receptor, and NMDA receptor have been explored as potential means to manage pain [12, 35, 36]. The motor coordination indicated by the rotarod performance test was not affected by the siRNA-GluN2B-induced analgesic effect as shown by Tan et al. [12]. All these suggest that siRNA is a feasible tool for precise, specific, and efficient “knock-down” of GluN2B, establishing a new option for NP therapy.

It has been documented that naked siRNA mediated downregulation of gene expression is transient and only lasts for 3 to 5 days [14]; hence, the lentivirus that is capable of expressing the target gene for several months and suitable for chronic pain treatment [15, 36] was introduced as a vehicle for siRNA. As hypothesized, results showed that after intrathecal injection the antinociception effect lasted for about 35 days, which provided a suitable tool for the treatment of chronic diseases including but not limited to NP. In addition, plasmid-expressing GFP was introduced into the Lv-si GluN2B viral system for tracking. Previous studies have found that the GluN2B subunit has a relatively restricted distribution in pain regulatory pathways, such as in the superficial dorsal horn of the spinal cord [37]. Our results showed that the lentivirus was successfully transfected into dorsal horn of CCI rats, which was consistent with the location of GluN2B. Results also showed the GluN2B expression along with the pain threshold significantly decreased after the Lv-siGluN2B injection, suggesting that Lv-siGluN2B may suppress nociception in rats of the NP model through decreasing GluN2B subunit expression.

Motor behavior and other side effects caused by the decreasing GluN2B were not examined in this study. It is imperative to investigate all possible effects caused by the lentivirus before preclinical studies. Additionally, in clinical practice, the levels and timing of GluN2B expression should be controlled precisely; thus, an inducible gene expression system such as an RU486 regulating system is necessary. Further studies are warranted.

In summary, results in the present study demonstrate that an intrathecal injection of Lv-siGluN2B significantly and continuously attenuates the nociception of CCI rats. These findings suggest that Lv-siGluN2B represents a new potential option for NP management.
